# Make Me Want to Pay. A Three-Way Interaction Between Procedural Justice, Distributive Justice, and Power on Voluntary Tax Compliance

**DOI:** 10.3389/fpsyg.2019.01632

**Published:** 2019-07-12

**Authors:** Marius van Dijke, Lemessa Bayissa Gobena, Peter Verboon

**Affiliations:** ^1^Rotterdam School of Management, Erasmus University, Rotterdam, Netherlands; ^2^Nottingham Business School, Nottingham Trent University, Nottingham, United Kingdom; ^3^College of Finance, Management and Development, Ethiopian Civil Service University, Addis Ababa, Ethiopia; ^4^Faculty of Psychology and Educational Sciences, Open University of the Netherlands, Heerlen, Netherlands

**Keywords:** distributive justice, procedural justice, power, voluntary tax compliance, deterrence

## Abstract

Tax compliance involves a decision where personal benefits come at the expense of society and its members. We explored the roles of procedural and distributive justice and citizens’ perceptions of the tax authority’s power in stimulating voluntary tax compliance. Distributive and procedural justice have often (but not always) been shown to interact in such a way that high distributive justice *or* high procedural justice is sufficient to predict positive responses to authorities and the social collective they represent. We examined whether this interaction predicts voluntary (but not enforced) tax compliance, in particular among citizens who perceive the tax authority’s power as high (vs. low). The results of two field studies among Ethiopian (Study 1) and United States (Study 2) taxpayers supported our predictions. With this research we connect the roles of two core social psychological antecedents of tax compliance (i.e., distributive and procedural justice) with that of a deterrent factor (i.e., authority power) and obtain support for the psychological process underlying the Distributive Justice × Procedural Justice interaction in two diverging tax environments.

## Introduction

Complying with tax laws and regulations involves a dilemma where personal gains that result from non-compliance come at a cost for society and its members (Kaplan et al., 1997; Chung and Trivedi, 2003; McGee, 2006; Maciejovsky et al., 2012; Molero and Pujol, 2012; Dowling, 2014; Gangl et al., 2015). This dilemma facing taxpayers has captured the attention of scholars from various disciplines for at least half a century (see Kirchler, 2007 for an overview). As part of their scientific investigation into tax compliance, social psychologists have identified various factors that predict the decision to voluntarily comply with taxation laws and regulations (i.e., voluntary tax compliance). Two of the most important antecedents of voluntary tax compliance that research has identified are the distributive justice of the tax authority (e.g., Cowell, 1992; Wenzel, 2002, 2003; Saad, 2011) and the procedural justice of this authority (Hartner et al., 2008; Farrar, 2015).

Surprisingly, very few researchers have considered if (and when) these two justice dimensions may *interact* to predict voluntary tax compliance (see Farrar and Thorne, 2012, for the only exception that we know of). This is despite the investigation of the Distributive Justice × Procedural Justice interaction effect on responses to authorities (e.g., trust in the authority; Brockner et al., 1995) and social systems (e.g., organizational commitment; Brockner et al., 1994) in social and applied psychology (see Brockner and Wiesenfeld, 1996 for an overview). Specifically, the interactive effect of distributive and procedural justice on responses to authorities and the social collectives that they represent often takes a form in which high distributive justice *or* high procedural justice is enough to lead to constructive responses to the authority or the collective (i.e., a substituting interaction). Put differently, less constructive responses are most likely to result when distributive justice *and* procedural justice are both low. In the present research we test if distributive and procedural justice interact in a substituting way to predict voluntary tax compliance.

More important from a theoretical perspective, we identify a novel, theoretically relevant moderator of the Distributive Justice × Procedural Justice interaction on voluntary tax compliance. Specifically, we argue that distributive and procedural justice interact in a substituting way to predict voluntary compliance more strongly when the authority’s power is perceived as being high, rather than low. Although the majority of prior research that studied procedural and distributive justice simultaneously has obtained the above described substituting interaction (Brockner and Wiesenfeld, 1996), some studies failed to reveal it (Dipboye and de Pontbriand, 1981; Tyler and Caine, 1981; De Cremer et al., 2010; Bianchi et al., 2015). In fact, in the only study that tested if distributive and procedural justice interactively influence voluntary tax compliance, Farrar and Thorne (2012) found no evidence for this prediction. By focusing on the moderating role of authority power (and effectively testing a three-way interaction between authority power, distributive justice, and procedural justice), the present research aims to identify *when* the Distributive Justice × Procedural Justice interaction is more (vs. less) likely to predict voluntary tax compliance.

Furthermore, by identifying authority power as a relevant moderator of the Distributive Justice × Procedural Justice interaction on voluntary tax compliance, our research presents a direct test of the process that drives this interaction effect (Spencer et al., 2005; MacKinnon and Fairchild, 2009; Jacoby and Sassenberg, 2011). The Distributive Justice × Procedural Justice interaction effect on responses to authorities and the collective is often explained as resulting from a concern to assess whether authorities will abuse their power (Chen et al., 2003; Blader and Chen, 2011; Bianchi et al., 2015). Such concerns of power abuse should be less salient when the authority has low (rather than high) power. This is why we expect that the Distributive Justice × Procedural Justice interaction predicts voluntary compliance more strongly when the authority’s power is perceived as high, rather than low.

This research makes three contributions to the literature. First, taxpayers often perceive distributive justice as low. They may, for instance, feel that they do not enjoy the benefits of tax-funded public goods and services as much as they think they deserve, or they may view that they pay too much in taxes, while receiving insufficient returns on their tax money compared with other taxpayers (Alm et al., 1993; Trivedi et al., 2003; Wenzel, 2003; Bobek et al., 2007; Saad, 2011; Fjeldstad et al., 2012; Alon and Hageman, 2013; Dowling, 2014). We test if high (vs. low) procedural justice may counteract or buffer the undermining effect of low (vs. high) distributive justice on voluntary tax compliance. Second, the interaction between distributive and procedural justice has been shortlisted as a highly relevant conceptual and empirical contribution to the justice literature (Colquitt et al., 2005). By identifying a novel boundary condition – citizens’ perceptions of the tax authority’s power – to its effectiveness, this research clarifies *why* these two types of justice interact. Third, almost all tax compliance research has focused on developed nations (i.e., the United States, Europe, and Australia), with developing countries being neglected. Yet, tax environments in developing countries differ from those in developed nations. In developing countries taxpayers tend to view paying taxes as a burden, rather than a contribution to a common good, and authorities have low trust in taxpayers (i.e., “cops and robbers” taxation environments; Kirchler et al., 2008; Asaminew, 2010; Gobena and van Dijke, 2016). In developed countries, relations between taxpayers and tax authorities are often more harmonious (Hansen et al., 1992; Hume et al., 1999; Trivedi et al., 2003; McGee, 2006; Alm and Torgler, 2011; Maciejovsky et al., 2012; Molero and Pujol, 2012; Alon and Hageman, 2013; Bobek et al., 2013). We contribute to the ecological validity of tax compliance research by testing our predictions in both a developed and a developing country (i.e., the United States and Ethiopia).

## Theoretical Background and Hypothesis Development

### Distributive Justice, Procedural Justice, and Voluntary Tax Compliance

Distributive justice refers to the extent to which outcomes of a process that distributes rewards and burdens are perceived as matching implicit norms such as the equity rule (Adams, 1965; Thibaut and Walker, 1975; Verboon and van Dijke, 2007). The equity rule requires that individuals should receive benefits proportional to their contributions. Research has shown that individuals react in more positive ways when they perceive decision outcomes as fair (vs. unfair; e.g., Brockner, 2002; Bianchi et al., 2015). More specific to tax compliance research, studies have shown that citizens who perceive the distribution of tax burdens and benefits across individuals, groups, and society as a whole as fair show as a result more willing to voluntarily comply with tax laws and regulations (e.g., Wenzel, 2002, 2003; Verboon and Goslinga, 2009; Saad, 2011).

Focusing on distributive justice is insufficient to understand the behavior of members of social collectives. In particular, it is also relevant to consider the fairness of the procedures that are applied by authorities in enacting rules, resolving disputes, and allocating resources (i.e., procedural justice; Thibaut and Walker, 1975; Leventhal, 1980). Various factors affect the perceived fairness of procedures. Some of these factors include consistent application of the procedures across time, absence of decision-makers’ self-interest in the process, decisions being based on accurate information, and allowing decision recipients to voice their opinions in the decision-making processes (Thibaut and Walker, 1975; Leventhal, 1980; van den Bos et al., 1996). Authorities’ procedural justice is known to beget positive attitudes and cooperative behaviors from followers (Thibaut and Walker, 1975; Tyler and Blader, 2000). Some examples of such effects of procedural justice in various settings include rule-following (e.g., Tyler, 2009), and public support for police (e.g., Jason and Tyler, 2003). Tax compliance studies also show that citizens who perceive decisions enacted by the tax authority as high in procedural justice show as a result an increased willingness to voluntary compliance with tax regulations and laws (Hartner et al., 2008; Murphy and Tyler, 2008; Farrar, 2015).

However, scholars have recognized that distributive justice and procedural justice should not be studied in isolation, but rather as interactive predictors of responses to authorities and the system they represent (e.g., Brockner and Wiesenfeld, 1996). The often-obtained Distributive Justice × Procedural Justice interaction has been interpreted in different ways. Some researchers emphasized that procedural justice more strongly influences peoples’ reactions to a decision when distributive justice is low (vs. high; e.g., Shapiro, 1991). Others emphasized that distributive justice more strongly predicts individuals’ reactions when procedural justice is low (vs. high; e.g., Brockner et al., 1994). Both ways of zooming in on the Distributive Justice × Procedural Justice interaction imply, as noted, that high distributive justice or high procedural justice is sufficient to produce constructive responses to the authority or the social collective. Put differently, negative responses are most likely to result when distributive justice *and* procedural justice are both low.

The effect of the Distributive Justice × Procedural Justice interaction on responses to authorities and the collective they represent is often explained as resulting from a concern about possible abuse of power by the enacting authority (Chen et al., 2003; Blader and Chen, 2011; Bianchi et al., 2015). To evaluate the likelihood that the authorities will abuse their power, people examine distributive justice as well as procedural justice. High distributive or high procedural justice is generally sufficient to lead people to believe that the authority will not abuse his/her power. As a result, the presence of high distributive justice or high procedural justice is enough to promote constructive reactions toward the authority or the collective.

### The Role of Power

In the tax compliance context, power of the tax authorities is defined as “tax authorities’ capacity to detect and punish tax crimes” (Wahl et al., 2010, p. 385; see also Kirchler et al., 2008). Empirical research on the effects of the power of tax authorities focused on detection probabilities (e.g., Phillips, 2014), fines (e.g., Cebula, 2014), and audits (e.g., Bernasconi et al., 2014).

In this paper, we argue that the power of the tax authority is a meaningful element of the process underlying the effect of the Distributive Justice × Procedural Justice interaction on voluntary tax compliance. As noted, the effect of the Distributive Justice × Procedural Justice interaction on responses to authorities and the collective is often explained as resulting from a concern to assess whether the authority will abuse his or her power (Chen et al., 2003; Blader and Chen, 2011; Bianchi et al., 2015). Specifically, to make sense of whether the authority can be trusted not to abuse power, people examine distributive justice as well as procedural justice. As noted, high distributive *or* high procedural justice is generally sufficient to make people believe that the authority will not abuse his/her power. Therefore, we expect that the Distributive Justice × Procedural Justice interaction materializes in particular when the enacting authority’s power is perceived as high (vs. low). This results because citizens who perceive the authority’s power as high will have salient concerns about the authority abusing his/her power. When the authority’s power is low, such concerns are less likely to be salient.

As first support for this argument, Chen et al. (2003) and Blader and Chen (2011) found that the typically observed interaction between distributive and procedural justice where high distributive or high procedural justice is enough to produce constructive responses is not found in relationships where justice is enacted by a partner of lower status or rank. Unfortunately, it is not clear whether this effect resulted from fear of power abuse, because rank differences can be based on a number of variables besides power, most notably status, which is only modestly related with power (Magee and Galinsky, 2008; Anderson and Brown, 2010). In the present research we offer a direct test of the idea that the Distributive Justice × Procedural Justice interaction results from concerns about power abuse by considering the power of the authority as moderator of this interaction. In sum, the above argument results in our hypothesis:

The tax authority’s power moderates the substituting interaction effect of distributive and procedural justice of the tax authority on voluntary tax compliance, such that the Distributive Justice × Procedural Justice interaction will be pronounced when the authority’s power is high (vs. low).

## Overview of Studies

We tested our hypothesis in two surveys. For Study 1 we collected data from working professionals in the Ethiopian capital, Addis Ababa. We used existing, validated scales for all the variables of our study. In Study 2 we obtained data from United States taxpayers. Our hypothesis (which we tested in both studies) concerns the prediction of voluntary tax compliance by the interaction between distributive and procedural justice (as further moderated by authority power). For discriminant validity purposes, we therefore also included enforced tax compliance. Enforced tax compliance describes the extent to which citizens comply with tax rules and regulations because they feel forced to do so (i.e., out of fear of being punished upon non-compliance; Kirchler et al., 2008; Gangl et al., 2015; Gobena and van Dijke, 2016). Neither the Distributive Justice × Procedural Justice interaction nor the Distributive Justice × Procedural Justice × Authority Power interaction should predict enforced tax compliance.

### Study 1

#### Method

##### Respondents

We gathered data from 273 working professionals in the Ethiopian capital, Addis Ababa, over 3 months (March–May, 2016). Of the respondents, 88% were male and 12% were female. As for their age categories, 41% were between 20 and 30 years, 41% between 31 and 40, 16% between 41 and 50, 1% between 51 and 60, and 1% above 60. One percent of the respondents had completed primary education only, 5% had an associate degree, 52% had a bachelor’s degree, 35% had a master’s degree, and 7% had completed a Ph.D. Thirty one percent earned annually between 20,000 and 40,000 Ethiopian Birr, 25% earned between 40,000 and 60,000 Birr, 8% earned between 60,000 and 80,000 Birr, 20% earned between 80,000 and 100,000 Birr, 5% earned between 100,000 and 120,000 Birr, and 11% earned more than 120,000 Birr. (1 Birr is about $ 0.05). Asked about their experiences with the tax authority, 7% of the respondents reported to have had fewer than 2 years of experience with the tax authority, 20% had between 2 and 6 years, 35% had between 6 and 10 years, 29% had between 10 and 20 years, and 9% had more than 20 years of experience. With regard to their ethnicity, 29% of our respondents reported being Amhara, 16% as Tigray, 34% as Oromo, 3% as Gurage, and 18% as “other.”

##### Procedure

We distributed 487 printed questionnaires to respondents. We also included a cover letter and a postage-paid envelope for returning the filled-out questionnaire. In the cover letter we assured strict anonymity of responses and we explained the study purpose. With a few lagging respondents, assistant data collectors repeatedly made visits to their offices, and made phone calls to remind them of the questionnaire (to ensure a reasonable response rate). The role of the assistant data collectors was restricted to transferring enclosed, filled-in questionnaires to the researchers; they could in no way endanger the anonymity of the respondents. We received 284 filled out questionnaires (a response rate of 58%). We removed eleven respondents from the data set because they had skipped a significant number of items. Consequently, we included data from 273 respondents in the analyses. The data is available as Supplementary Material.

##### Measures

All items that we used in this study are included in Appendix. We used a 5-item scale from Verboon and van Dijke (2007) to measure the tax authority’s *distributive justice*. Item examples are “The use I make of all kinds of social services reflects in a proper way the taxes I pay” and “Regarding social services I get little return for my tax money” (reverse coded) (*1 = strongly disagree, 7 = strongly agree*). We combined these items into an index of distributive justice.

We measured *procedural justice* with Colquitt’s (2001) 7-item scale. This scale was adapted to the tax compliance context by Gobena and van Dijke (2016, 2017). Item examples (preceded by the stem “The following items refer to the procedures used to arrive at tax-related decisions.”) are “Those procedures have been based on accurate information” and “Those procedures have been free of bias” *(1 = strongly disagree, 7 = strongly agree)*. We combined these items into an index of procedural justice.

We used a 5-item scale from Kastlunger et al. (2013) to measure the tax authority’s *power* (see Verboon and van Dijke, 2007; Siglé et al., 2018, for similar measures of tax authority’s power). Item examples are “Tax evasion is likely to be detected” and “Tax authorities control frequently and profoundly.” *(1 = strongly disagree, 7 = strongly agree).* We these items into an authority power index.

We used a 5-item scale from Kirchler and Wahl (2010) to measure *voluntary tax compliance*. Item examples (preceded by the stem “When I pay my taxes as required by the Ethiopian tax laws and regulations, I do so…”) are “…because I like to contribute to everyone’s good” and “…because for me it’s the natural thing to do” *(1 = strongly disagree, 7 = strongly agree)*. We combined the items into an index of voluntary tax compliance.

We used a 5-item scale from Kirchler and Wahl (2010) to measure *enforced tax compliance*. Item examples (preceded by the stem “When I pay my taxes as required by the Ethiopian tax laws and regulations, I do so…”) are “…because I know that I will be audited” and “…because the punishments for tax evasion are very severe” *(1 = strongly disagree, 7 = strongly agree)*. We combined the items into an index of enforced tax compliance.

#### Results

Table 1 presents means, standard deviations, Cronbach alpha coefficients, and correlations between the study variables.

**TABLE 1:** Study 1 variables’ means, standard deviations, correlations, and reliabilities.

	**Mean (*SD*)**	**1**	**2**	**3**	**4**	**5**	**6**	**7**	**8**	**9**	
(1) Gender	0.12 (0.33)										
(2) Age range	1.79 (0.80)	–0.18^∗∗^									
(3) Education	4.42 (0.71)	–0.00	0.50^∗∗^								
(4) Annual income	2.75 (1.68)	–0.12	0.40^∗∗^	0.48^∗∗^							
(5) Years of experience	3.15 (1.06)	–0.17^∗∗^	0.75^∗∗^	0.45^∗∗^	0.37^∗∗^						
(6) Distributive justice	3.10 (1.18)	0.01	0.10	0.08	–0.05	0.11	0.70				
(7) Procedural justice	3.78 (1.43)	–0.01	0.01	0.04	−0.12^*^	0.02	0.34^∗∗^	0.93			
(8) Authority power	3.77 (1.34)	0.03	–0.01	0.08	–0.07	–0.04	0.54^∗∗^	0.14^*^	0.87		
(9) Enforced tax compliance	3.53 (1.48)	–0.01	–0.07	–0.11	–0.05	–0.08	0.20^∗∗^	0.08	0.03	0.80	
(10) Voluntary tax compliance	4.82 (1.49)	–0.03	0.01	0.04	–0.09	–0.03	0.45^∗∗^	0.42^∗∗^	0.22^∗∗^	–0.01	0.90

*N* = 273. Reliabilities (Cronbach’s α coefficients) are on the main diagonal for multi-item scales. ^*^p < 0.05, ^∗∗^*p* < 0.01.

Before testing our hypothesis, we conducted confirmatory factor analyses (CFA; Anderson and Gerbing, 1988; Bandalos and Finney, 2001). First, we estimated a 1-factor model. Next, we estimated a 5-factor model (voluntary tax compliance, enforced tax compliance, distributive justice, procedural justice, and power). Finally, we fitted a 6-factor model, which was identical to the 5-factor model, apart from the inclusion of a method factor that was uncorrelated to the other five factors (see Podsakoff et al., 2003). As fit indices we used the CFI, RMSEA, and PCFI. The fit of the 1-factor model was insufficient [χ^2^(325) = 2309.79, CFI = 0.52, RMSEA = 0.15 (90% CI = 0.144–0.156), PCFI = 0.48]. The fit of the 5-factor model was acceptable [χ^2^(318) = 642.74, CFI = 0.92, RMSEA = 0.061 (90% CI = 0.054–0.068), PCFI = 0.83] after allowing the error terms of item 1 and 2 of the procedural justice scale (see Appendix) to covary. This covariation of the first two procedural justice items reflects prior research, showing that the procedural justice scale has two components, that is, follower control (reflecting these two items) and leader benevolence (van Dijke and De Cremer, 2010). The 6-factor model also fitted the data well [χ^2^(292) = 518.71, CFI = 0.95, RMSEA = 0.053 (90% CI = 0.046–0.061), PCFI = 0.78]. According to CFI and RMSEA, the fit of the 6-factor model is slightly superior to that of the 5-factor model. Yet, the PCFI for this model is clearly lower than for the 5-factor model and, in fact, below the accepted threshold of 0.80 (Byrne, 2016). Thus, the CFAs support the validity of our specified measurement model. In fact, even if we accept the weak evidence for common method variance from the 6-factor model, this does not preclude the testing of our hypothesis, as it concerns an interaction effect, in which common method variance plays no explanatory role (Evans, 1985).

We tested our hypothesis with ordinary least squares (OLS) regression analyses. We entered the main effects of the predictor variables in Step 1. In step 2 we entered the two-way interactions between these variables. In Step 3 we entered the three-way interaction between these variables. (We standardize the predictor variables before calculating the interaction terms.) The results are presented in Table 2.

**TABLE 2:** Regression results of study 1.

**Dependent variable**	**Voluntary tax compliance**	**Enforced tax compliance**
**Step 1, *R^2^, R^2^_*adj*_***	0.28^∗∗∗^, 0.27^∗∗∗^	0.05^∗∗^, 0.04^∗∗^
Distributive justice	0.30(5.50)***	0.01(0.16)
Procedural justice	0.34(5.29)^∗∗∗^	0.25(3.33)^∗^
Authority power	−0.004(−0.06)	−0.10(−1.46)
**Step 2, *R^2^, R^2^_*adj*_, R^2^_*change*_***	0.29, 0.27, 0.01	0.06, 0.03, 0.01
Distributive justice	0.31(5.54)^∗∗∗^	0.002(0.03)
Procedural justice	0.33(4.87)^∗∗∗^	0.27(3.57)^∗∗∗^
Authority power	−0.003(−0.05)	−0.11(−1.53)
Distributive justice × Procedural justice	−0.09(−1.37)	0.12(1.57)
Distributive justice × Authority power	0.05(0.81)	−0.07(−0.92)
Procedural justice × Authority power	0.05(0.90)	−0.02(−0.38)
**Step 3, *R^2^, R^2^_*adj*_, R^2^_*change*_***	0.30^*^, 0.29^*^, 0.02^*^	0.06, 0.03, 0.001
Procedural justice	0.31(4.62)^∗∗∗^	0.27(3.49)^∗^
Distributive justice	0.39(6.14)^∗∗∗^	0.02(0.24)
Authority power	0.04(0.55)	−0.10(−1.38)
Distributive justice × Procedural justice	−0.12(−1.88)	0.11(1.44)
Distributive justice × Authority power	0.06(0.94)	−0.07(−0.89)
Procedural justice × Authority power	0.02(0.31)	−0.03(−0.48)
Distributive justice × Procedural justice × Authority power	−0.16(−2.55)^∗^	−0.03(−0.46)

*N* = 273; Table presents standardized β coefficients and *t*-values in brackets; ^*^p < 0.05, ^∗∗^*p* < 0.01, ^∗∗∗^*p* < 0.001.

As shown in Table 2, consistent with prior findings, distributive and procedural justice, significantly predicted voluntary tax compliance; the main effect of authority power on voluntary tax compliance was not significant. The Distributive Justice × Procedural Justice interaction was marginally significant on voluntary tax compliance (*p* = 0.06).

**FIGURE 1:**
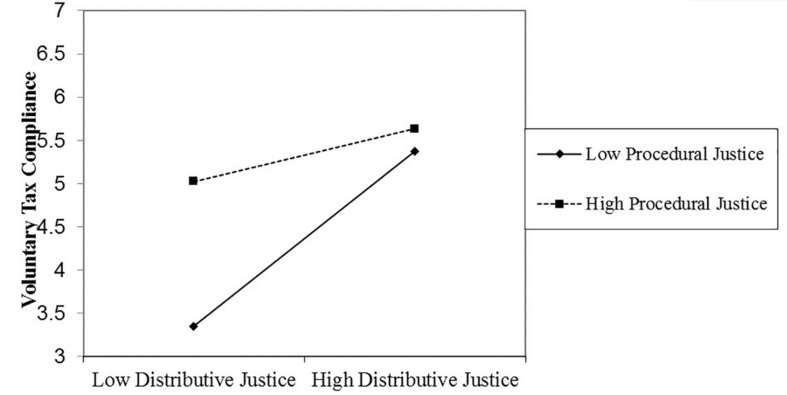
The significant distributive justice × procedural justice interaction effect on voluntary tax compliance when authority power is high (Study 1).

**FIGURE 2:**
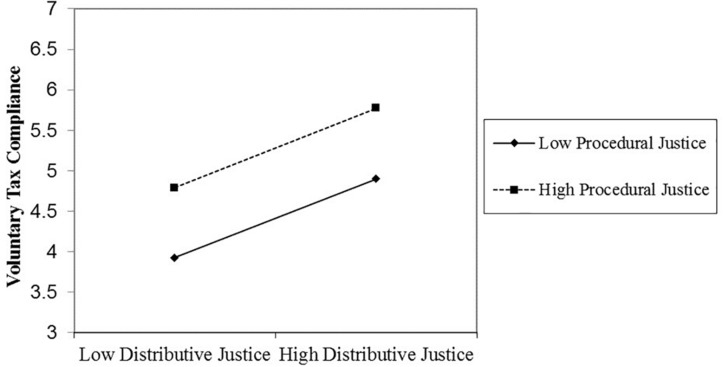
The non-significant distributive justice × procedural justice interaction effect on voluntary tax compliance when authority power is low (Study 1).

In line with our hypothesis, in step 3, the three-way interaction between procedural justice, distributive justice, and power was significant. This interaction is depicted in Figures 1, 2. We used simple slopes analyses to decompose this interaction (Aiken and West, 1991). We conducted these analyses using the PROCESS macro for SPSS (Model 3; Hayes, 2012). These analyses revealed that the Distributive Justice × Procedural Justice interaction significantly predicted voluntary tax compliance when the tax authority’s power was high (1 *SD* above the mean; β = −0.35, *t* = −2.77, *p* = 0.01) but not when it was low (1 *SD* below the mean; β = 0.002, *t* = 0.02, *p* = 0.99).

Because the two-way Distributive Justice × Procedural Justice interaction was significantly related to voluntary tax compliance when authority power was high, we subsequently conducted simple slopes tests of the simple main effects. These analyses revealed that when authority power was high (1 *SD* above the mean) and procedural justice was low (1 *SD* below the mean), distributive justice significantly predicted voluntary tax compliance (β = 0.44, *t* = 4.67, *p* < 0.01). When power was high (1 *SD* above the mean) and procedural justice was high (1 *SD* above the mean), distributive justice also significantly predicted voluntary tax compliance, but the relationship was clearly weaker (β = 0.20, *t* = 2.27, *p* = 0.02).

However, our argument implies that when the tax authority’s power is high, high procedural or high distributive justice is sufficient to produce high levels of voluntary tax compliance. Figure 1 appears to be in line with this predicted pattern. To formally test this pattern, we tested the simple slopes of procedural justice on voluntary tax compliance when distributive justice was high (vs. low) and when the tax authority’s power was high (vs. low). These analyses showed that the tax authority’s power was high (1 *SD* above the mean) and distributive justice was low (1 *SD* below the mean), procedural justice significantly predicted voluntary tax compliance (β = 0.56, *t* = 5.40, *p* < 0.01). When authority power was high (1 *SD* above the mean) and distributive justice was high (1 *SD* above the mean), procedural justice did not significantly predict voluntary tax compliance was not significant (β = 0.09, *t* = 0.62, *p* = 0.53).

These results indicate that procedural justice and distributive justice interact to predict voluntary tax compliance, such that for high voluntary tax compliance it is enough that procedural or distributive justice is high. But this interaction is restricted to citizens who view the tax authority as having high power. In further evidence of our argument, Table 2 also shows that distributive justice, procedural justice, and the tax authority’s power did not interact to predict enforced tax compliance.

#### Discussion

Study 1 presents first evidence in support of our hypothesis. However, we collected the data in one specific tax climate, that is, among Ethiopian taxpayers. Ethiopia is characterized by a tense connection between the tax authority and taxpayers (Bekana et al., 2014; Gobena and van Dijke, 2016). To test the generalizability of our findings, in Study 2 we sought to replicate our findings obtained in the Ethiopian context in a taxation climate where the relationship between taxpayers and the tax authority is friendlier, that is, among United States income taxpayers.

### Study 2

#### Method

We used Amazon’s Mechanical Turk (AMT) to recruit participants. AMT offers online access to a large pool of respondents, which makes data collection faster and inexpensive (Buhrmester et al., 2011). AMT is widely used to gather data across a wide range of the social sciences (Buhrmester et al., 2011; Goodman, 2013). Studies that have employed AMT cover topics as diverse as procedural justice (van Dijke et al., 2015) and acting professionally (Uhlmann et al., 2013). Previous tax compliance research has also relied on AMT to collect data (Gobena and van Dijke, 2017). The reliability of data collected via AMT for both survey and experimental studies mirrors (and sometimes is even superior to) that of data obtained using traditional methods (Behrend et al., 2011; Casler et al., 2013; Holden et al., 2013; Bartneck, 2015).

##### Sample and procedure

We invited respondents who currently had work that earned them taxable income and hence had experience with the tax authority to participate in the study. There were no missing values because all 248 respondents that we recruited for the study responded to all items. The data is available as Supplementary Material. We informed respondents that the study was being about “individuals’ interactions with authorities.” Of the 248 respondents, 48% were male, and 52% were female. Thirty percent were between 20 and 30 years of age, 30% between 31 and 40, 18% between 41 and 50, 12% between 51 and 60, and 10% above 60 years of age. Twenty-four percent had a high school diploma, 15% had completed vocational education, 46% had a bachelor’s degree, 10% had completed a master’s degree, and 4% had completed a Ph.D. Eighteen percent the respondents earned annually less than 20,000 USD, 13% earned between 20,000 and 29,999 USD, 20% earned between 30,000 and 39,999 USD, 13% earned between 40,000 and 49,999 USD, and 36% earned 50,000 USD or more. Six percent of respondents had fewer than 2 years of experience with the tax authority; 17% had between 2 and 6 years of experience; 14% had between 6 and 10 years; 27% had between 10 and 20 years; and 36% had more than 20 years of experience. Eighty-eight percent identified themselves as White/Caucasian, 4% as Hispanic American, 4% as African American, 2% as Asian American, 1% as Native American, and 1% as having an “other” ethnic background.

##### Measures

We measured all study variables (i.e., procedural justice, distributive justice, authority power, and voluntary tax compliance) with the same instruments as in Study 1 (see Appendix), except for some wording changes in which “Ethiopia” was replaced by “the United States.” All variables were measured on a 7-point Likert scale (*1 = strongly disagree, 7 = strongly agree*).

#### Results

Table 3 presents means, standard deviations, Cronbach’s alpha coefficients, and correlations between the study variables.

**TABLE 3:** Study 2 variables’ means, standard deviations, correlations, and reliabilities.

	**Mean (*SD*)**	**1**	**2**	**3**	**4**	**5**	**6**	**7**	**8**	**9**	**10**	**11**
(1) Gender	0.52 (0.50)											
(2) Age range	2.41 (1.29)	0.71										
(3) Education	3.54 (1.11)	–0.08	–0.02									
(4) Annual income	3.37 (1.52)	–0.42	0.15^*^	0.27^∗∗^								
(5) Years of experience	3.70 (1.27)	0.01	0.71^∗∗^	0.07	0.25^∗∗^							
(6) Distributive justice	3.14 (1.20)	0.02	−0.13^*^	0.11	–0.09	−0.15^*^	0.76					
(7) Procedural Justice	3.85 (1.38)	0.01	−0.14^*^	0.05	–0.06	−0.16^*^	0.45^∗∗^	0.93				
(8) Authority power	3.85 (1.37)	0.15^*^	–0.21^∗∗^	–0.18^∗∗^	–0.06	–0.21^∗∗^	0.53^∗∗^	0.18^∗∗^	0.89			
(9) Enforced tax compliance	4.11 (1.40)	0.06	–0.09	–0.08	0.08	−0.16^*^	–0.04	–0.26^∗∗^	0.19^∗∗^	0.39^∗∗^	0.84	
(10) Voluntary tax compliance	4.83 (1.50)	0.14^*^	0.03	0.10	–0.08	–0.01	0.54^∗∗^	0.47^∗∗^	0.24^∗∗^	–0.43^∗∗^	–0.17^∗∗^	0.90

*N* = 248. Reliabilities (Cronbach’s α coefficients) are on the main diagonal for multi-item scales. ^*^p < 0.05, ^∗∗^*p* < 0.01.

Like in Study 1, we conducted CFAs. The 1-factor model showed insufficient fit [χ^2^(325) = 2543.84, CFI = 0.49, RMSEA = 0.166 (90% CI = 0.160–0.172), PCFI = 0.45]. The 5-factor model showed acceptable fit [χ^2^(318) = 774.42, CFI = 0.90, RMSEA = 0.076 (90% CI = 0.069–0.083), PCFI = 0.81], when the error terms of item 1 and 2 of the procedural justice scale were allowed to covary. The 6-factor model also showed acceptable fit [χ^2^(292) = 650.98, CFI = 0.92, RMSEA = 0.071 (90% CI = 0.063–0.078), PCFI = 0.76]. Thus, as in Study 1, although the CFI and RMSEA indicate a marginally better fit for the 6-factor model, the PCFI for of this model was lower than for the 5-factor model. It was also below the accepted threshold of 0.80. In sum, the CFAs in this study also support the validity of our specified measurement model.

We tested our hypothesis using OLS regression, as we did in Study 1. We entered the main effects of distributive justice, procedural justice, and authority power, and their interactions, in the same way as we did in Study 1. The results are presented in Table 4.

**TABLE 4:** Regression results of study 2.

**Dependent variable**	**Voluntary tax compliance**	**Enforced tax compliance**
**Step 1, *R^2^, R^2^_*adj*_***	0.36^∗∗∗^, 0.35^∗∗∗^	0.13^∗∗∗^, 0.12^∗∗∗^
Distributive justice	0.29(5.05)^∗∗∗^	−0.29(−4.30)^∗∗∗^
Procedural justice	0.43(6.47)^∗∗∗^	−0.06(−0.81)
Authority power	−0.05(−0.79)	0.28(3.94)^∗∗∗^
**Step 2, *R^2^, R^2^_adj_, R^2^_change_***	0.36, 0.35, 0.01	0.14, 0.12, 0.01
Distributive justice	0.30(5.13)^∗∗∗^	−0.28(−4.10)^∗∗∗^
Procedural justice	0.41(5.90)^∗∗∗^	−0.06(−0.69)
Authority power	−0.04(−0.60)	0.28(3.84)^∗∗∗^
Distributive justice × Procedural justice	−0.08(−1.14)	0.06(0.72)
Distributive justice × Authority power	0.09(1.25)	0.02(0.25)
Procedural justice × Authority power	−0.02(−0.37)	−0.03(−0.44)
**Step 3, *R^2^, R^2^_adj_, R^2^_change_***	0.37^*^, 0.36^*^, 0.01^*^	0.15, 0.12, 0.01
Distributive justice	0.36(5.59)^∗∗∗^	−0.22(−2.97)^∗^
Procedural justice	0.40(5.83)^∗∗∗^	−0.06(−0.77)
Power	0.00(−0.01)	0.31(4.16)^∗∗∗^
Distributive justice × Procedural justice	−0.09(−1.36)	0.04(0.55)
Distributive justice × Authority power	0.09(1.36)	0.03(0.33)
Procedural justice × Authority power	−0.02(−0.39)	−0.03(−0.45)
Distributive justice × Procedural justice × Authority power	−0.13(−2.16)^∗^	−0.12(−1.68)

*N* = 248; Table presents standardized β coefficients and *t*-values in brackets; ^*^p < 0.05, ^∗∗^*p* < 0.01, and ^∗∗∗^*p* < 0.001.

As shown in Table 4, consistent with Study 1, distributive and procedural justice significantly predicted voluntary tax compliance; the main effect of authority power did not significantly predict voluntary tax compliance. Furthermore, the Distributive Justice × Procedural Justice interaction was not significant (*p* = 0.22), although it was in the expected direction.

**FIGURE 3:**
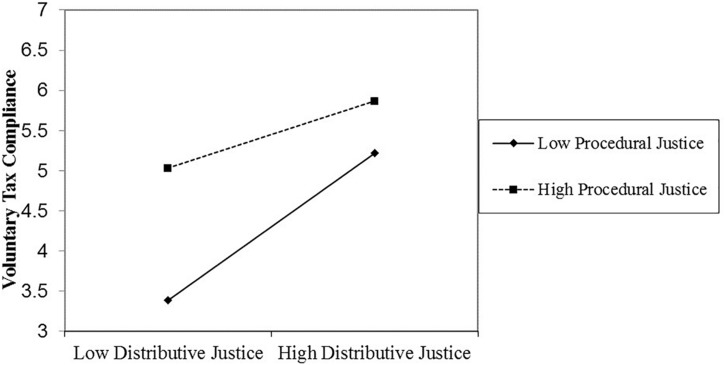
The significant distributive justice × procedural justice interaction effect on voluntary tax compliance when authority power is high (Study 2).

**FIGURE 4:**
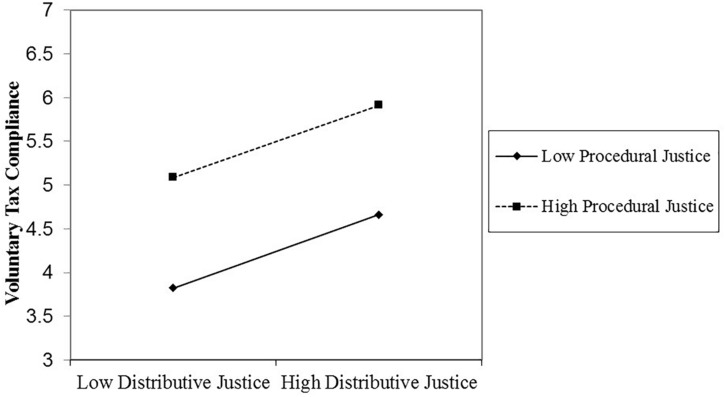
The non-significant distributive justice × procedural justice interaction effect on voluntary tax compliance when authority power is low (Study 2).

Similar to Study 1, in support of our hypothesis, the three-way interaction between distributive justice, procedural justice, and the authority’s power was significant. Figures 3, 4 visually depict the shape of this interaction. We proceeded to decompose this interaction with simple slopes analyses (Aiken and West, 1991), again using the PROCESS macro for SPSS (model 3; Hayes, 2012). These analyses showed that when the authority’s power was high (1 *SD* above the mean), the Distributive Justice × Procedural Justice interaction significantly predicted voluntary tax compliance (β = −0.18, *t* = −2.19, *p* = 0.03). However, when the authority’s power was low (1 *SD* below the mean), the Distributive Justice × Procedural Justice interaction did not significantly predict voluntary tax compliance (β = −0.003, *t* = −0.04, *p* = 0.97).

Because the simple Distributive Justice × Procedural Justice interaction significantly predicted voluntary tax compliance among citizens who perceived the tax authority to wield high power but not among those who perceived the tax authority to wield low power, we proceeded, as in Study 1, with further simple slopes tests in which we decomposed the Distributive Justice × Procedural Justice interaction among citizens who perceived the tax authority to wield high power. These analyses revealed that when the authority had high power (1 *SD* above the mean) and procedural justice was low (1 *SD* below the mean), distributive justice significantly predicted voluntary tax compliance (β = 0.61, *t* = 4.06, *p* < 0.01). When authority power was high (1 *SD* above the mean) and procedural justice was high (1 *SD* above the mean), distributive justice did not significantly predict voluntary tax compliance (β = 0.28, *t* = 3.19, *p* < 0.01).

As stated earlier, our argument implies that when power of the tax authority is high, high procedural or high distributive justice is enough to produce high levels of voluntary tax compliance. Similar to Figures 1, 2 appears to be in line with this predicted pattern. To formally test this pattern, we tested the simple slopes of procedural justice on voluntary tax compliance when distributive justice was high (vs. low), and when the power of the tax authority was perceived to be high (vs. low). These analyses revealed that when authority power was high (1 *SD* above the mean) and distributive justice was low (1 *SD* below the mean), procedural justice significantly predicted voluntary tax compliance (β = 0.55, *t* = 5.18, *p* < 0.01). When authority power was high (1 *SD* above the mean) and distributive justice was high (1 *SD* above the mean), procedural justice marginally significantly predicted voluntary tax compliance (β = 0.22, *t* = 1.71, *p* = 0.09).

As in Study 1, these results show that voluntary tax compliance is predicted by the Distributive Justice × Procedural Justice interaction, such that for high voluntary tax compliance, it is enough that distributive or procedural justice is high. But the Distributive Justice × Procedural Justice interaction is restricted to citizens who perceive the tax authority’s power as high (rather than low). Furthermore, distributive justice, procedural justice, and the tax authority’s power did not interact to predict enforced tax compliance.

## Supplemental Analyses

The Distributive Justice × Procedural Justice interaction marginally predicted voluntary tax compliance in Study 1 and non-significantly in Study 2. To assess if this interaction significantly predicts voluntary tax compliance across the two studies, we conducted a within-paper meta-analysis using MAVIS in the R Shiny package for R. We weighted the study effect sizes by the inverse variance and used the DerSimonian-Laird estimator for tau^2^ and Fisher’s *z* transformation of the correlations (i.e., derived from the β coefficients of the interaction; Hamilton et al., 2016). This analysis showed that across Studies 1–2 the Distributive Justice × Procedural Justice interaction significantly predicted voluntary tax compliance (*r* = −0.12; 95% CI [−0.21;−0.04], *z* = −2.79, *p* = 0.01).

## General Discussion

One important dilemma that individuals face is whether to comply with tax laws and regulations, where the personal gains that result from non-compliance come at a cost for society and its members. We tested in an Ethiopian (Study 1) and United States (Study 2) sample of income taxpayers if the Distributive Justice × Procedural Justice interaction (i.e., high distributive *or* high procedural justice being enough to produce voluntary compliance) is restricted to tax authorities with high (vs. low) power. We obtained this predicted three-way interaction only on voluntary, and not on enforced, tax compliance.

### Theoretical Implications

It has been argued that the Distributive Justice × Procedural Justice interaction, where high distributive or high procedural justice is enough to produce cooperative responses, results from concerns to assess whether one will be the victim of power abuse by the enacting authority. Based on this argument, we expected that the Distributive Justice × Procedural Justice interaction materializes in particular when the enacting authority’s power is high (vs. low). We expected this because in such situations, citizens are relatively likely to fear abuse. Previous work has shown that the typically observed Distributive Justice × Procedural Justice interaction where high distributive or high procedural justice is enough to produce cooperative responses, is not found when the person enacting procedural and distributive justice is of lower rank than the person on the receiving end of justice (Chen et al., 2003; Blader and Chen, 2011). However, it is not clear whether this effect resulted from fear of power abuse or from another process because rank differences can be based on a number of variables besides power, most notably status. And status is only modestly related with power (Magee and Galinsky, 2008; Anderson and Brown, 2010). Thus, our research adds to this prior work by offering a direct test of the idea that the Distributive Justice × Procedural Justice interaction pattern results from concerns about power abuse by authorities. Moreover, our design allows leaving the typical situation intact where justice is enacted by an authority of higher rank, such as the relationship between taxpayers and the tax authority, but also relationships between managers and employees at the work floor. In sum, identifying authority power as a novel, theoretically relevant boundary condition to the Distributive Justice × Procedural Justice interaction helps us understand better *when* and also *why* these two types of justice interact.

Furthermore, our cross-cultural studies with two samples that strongly differ in terms of tax climates serve the purpose of filling the void in studies that compare the voluntary tax compliance behavior of developed and developing countries (Gobena and van Dijke, 2016). Our study uniquely explores how social psychological and deterrent factors moderate each other in stimulating voluntary tax compliance across culturally different samples – one in Ethiopia and the other in the United States. Accordingly, we contribute to the ecological validity of integrative roles of social psychological and deterrence factors on tax compliance. Deterrent factors are those factors that force individuals to behave against their will; one of such factors is power wielded by authorities (Kirchler et al., 2008; Kastlunger et al., 2013). Deterrent factors belong to a distinct stream of research on tax compliance behavior that presumes that taxpayers’ compliance with taxation laws and rules depends on their self-interest and consequent comparison of the costs and benefits of evading taxes (Allingham and Sandmo, 1972). The deterrent line of research is based on the notion that taxpayers are selfish and will decide to pay taxes only when they believe the expected costs of evading taxes (i.e., tax audits and subsequent punishments) outweigh the benefits of evasion (i.e., money saved from unpaid taxes).

### Practical Implications

This research also offers contributions to the practice of tax administration. First, prior research has identified procedural and distributive justice as important antecedents of voluntary tax compliance (e.g., Wenzel, 2003; Saad, 2011). Unfortunately, taxpayers often perceive distributive justice as low, owing, for example, to the judgment of their exchange with the government as unfair, inequitable distribution of tax burdens and benefits, or simply because they view paying taxes as unfavorable, which taints distributive justice perceptions. We showed, however, that high (vs. low) procedural justice of the tax authorities buffers the effects of lowered perceived distributive justice. Therefore, tax authorities can stimulate a higher level of voluntary tax compliance by making their decision-making procedures free of their own self-interest, basing taxation decisions on accurate information, and letting the taxpayers voice their opinions in the decisions.

From a different vantage point, high (vs. low) distributive justice buffers the relationship between lowered procedural justice and voluntary tax compliance. This is also relevant to consider because it may often not be possible, for instance, to offer voice to taxpayers in taxation decisions, or to convince taxpayers that the tax authority used all relevant information to arrive at a decision. In such situations, high distributive justice is important to stimulate high levels of voluntary tax compliance.

However, our results also show that for either justice dimension to buffer effects of low scores on the other dimension, it is important to be also perceived as having high power (i.e., being capable of detecting and punishing non-compliance). Thus, even in antagonistic taxation climates, tax authorities are advised to gradually build power by increasing the percentage of tax evasion they detect and having convincing knowledge and competence to detect tax evasion.

### Limitations and Suggestions for Future Research

One limitation that should be discussed results concerns the fact that it is not possible to arrive at causal conclusions, due to the cross-sectional design that we used. Future research should draw unambiguous causal conclusions using longitudinal or experimental designs. Yet, we note that prior experimental research has shown that high (vs. low) procedural justice increases voluntary tax compliance (e.g., Wenzel, 2006; Doyle et al., 2009; van Dijke and Verboon, 2010). Furthermore, various experiments have identified the interactive effect of procedural and distributive justice on various outcomes that are related to compliance, such as voluntary cooperation in groups (Brockner and Wiesenfeld, 1996).

Another limitation is that we studied voluntary tax compliance (rather than actual levels of compliance) based on self-reported data. The use of self-reported measures in the tax compliance literature is common because it is very difficult to obtain tax compliance data from other sources (e.g., Wahl et al., 2010; Kastlunger et al., 2013; Gobena and van Dijke, 2016; Siglé et al., 2018). Moreover, one important limitation of objective tax compliance data is that it cannot detect various types of motives that underlie compliance; something that was of primary interest in our research (cf. van Dijke and Verboon, 2010). It should be noted that a number of studies found that self-reported compliance significantly predicts objective compliance (see e.g., Tittle, 1980; Hite, 1988; but see also Hessing et al., 1988).

Our measure of distributive justice (taken from Verboon and van Dijke, 2007) combines three elements of distributive justice that are in the literature sometimes more explicitly distinguished, specifically, horizontal equity, vertical equity, and exchange equity. Exchange equity refers to “the perceived value of tax-funded government benefits and services received relative to one’s tax contribution” (Wenzel, 2003, p. 44). This element is well-captured in several items that we used (see Appendix) including “The use I make of all kinds of social services reflects in a proper way the taxes I pay.” Vertical equity describes “the burden of taxes for certain social strata relative to other strata” (Wenzel, 2003, p. 44). This distributive justice element is most clearly reflected in the item “Some groups in society benefit more from the tax system than I do.” Finally, horizontal equity “concerns the burden of taxes for members relative to others within a given social stratum” (Wenzel, 2003, p. 44). This element of distributive justice is most clearly reflected in the item “I think it is not fair that some people pay less tax than me while they benefit equally from all amenities.” Future research should use instruments that distinguish these distributive justice elements and test if each element interacts with procedural justice and authority power similar to how the overall distributive justice scale interacts with these variables. Based on distributive justice theories such as equity theory (Adams, 1965), which is well-supported by research, we expect similar results for the three elements. Specifically, this theory argues that the three distributive justice elements are related to each other because citizens use social comparison information that is inherent to vertical and horizontal equity as input to evaluate exchange equity.

The measure of power that we used (taken from Kastlunger et al., 2013), which focuses on effectively detecting tax non-compliance, connects well with established definitions of power in the tax compliance literature (i.e., “tax authorities’ capacity to detect and punish tax crimes”; Wahl et al., 2010, p. 385; see also Kirchler et al., 2008; see Verboon and van Dijke, 2007; Siglé et al., 2018, for similar measures of power). Kastlunger et al. (2013) labeled this scale “legitimate power.” However, the tax compliance literature usually takes a much broader view of legitimate power as “based on the fact that the legitimate authorities use information, charisma, legitimization, and expertise to convince taxpayers that it is the right course of action to cooperate” (Gangl et al., 2015, p. 16). Thus, one way to view our results is that power as we measured it combined with procedural and/or distributive justice (which involves legitimization and offering information; Tyler, 2006) provides an encompassing index of the legitimacy of tax authorities. Future research should test if the three-way interaction that we obtained on voluntary tax compliance is mediated by legitimacy perceptions.

Finally, authority power moderated the interaction between distributive and procedural justice on voluntary tax compliance, but not lower-order effects of these two justice dimensions. This is in line with the argument for the Distributive Justice × Procedural Justice interaction that high distributive *or* high procedural justice is generally sufficient to induce the belief that the authority will not abuse his/her power. However, van Dijke et al. (2010) found that high (vs. low) procedural justice influenced trust in the authority and subsequent positive responses to the authority only among employees who interacted with an authority who was high (vs. low) in power. It should be noted that van Dijke et al. (2010) operationalized power different from how we did this. Specifically, they relied on theoretically derived scales of reward power (e.g., “My supervisor can increase my pay level”) and coercive power (“My supervisor can give me undesirable job assignments”; Hinkin and Schriesheim, 1989). Kastlunger et al. (2013) also developed a coercive power scale in the tax compliance context. This scale does not refer to power based upon the ability to reward or punish citizens, but to perceptions of authorities as being negative or hostile in their orientation toward tax payers (e.g., “Tax authorities primarily aim to punish” and “Tax authorities nurture hostile feelings toward taxpayers”). Given this focus, we decided not to use this scale in our research (but see Gobena and van Dijke, 2016, for evidence that Kastlunger et al.’s (2013) legitimate and coercive power scales interact with procedural justice to predict voluntary tax compliance among business owners). Future research should test if operationalizations of power other than the one that we used moderate the Distributive Justice × Procedural Justice interaction and lower-order effects of procedural and distributive justice on voluntary tax compliance.

## Conclusion

Distributive and procedural justice and justice have both been identified as factors that enhance voluntary tax compliance. We focused on the interactive effect of these two types of justice on voluntary tax compliance, identifying power of the tax authority as a boundary moderator of the interactive relationship. In taking this approach, we show that fair procedures can make up for the perception of unfair outcomes, as long as the tax authority’s power is high. Integrating these diverse factors that promote voluntary tax compliance in two extremely divergent tax climates has thus relevant theoretical and practical implications.

## Data Availability

All datasets generated for this study are included in the manuscript and/or the Supplementary Files.

## Ethics Statement

This study was carried out in accordance with the recommendations of the guidelines of the Internal Review Board of Rotterdam School of Management, Erasmus University, with written informed consent from all subjects. All subjects gave written informed consent in accordance with the Declaration of Helsinki. The protocol was approved by the Internal Review Board of Rotterdam School of Management, Erasmus University.

## Author Contributions

MvD collected the data for Study 2 and wrote the manuscript together with LG, who also conducted the analyses and collected the data for Study 1. PV helped in writing the manuscript and data analyses.

## Conflict of Interest Statement

The authors declare that the research was conducted in the absence of any commercial or financial relationships that could be construed as a potential conflict of interest.

## Appendix

Below is a list of two of the measures used in this paper. All responses were on a Likert scale (1 = *strongly disagree*, 2 = *disagree*, 3 = *moderately disagree*, 4 = *neither agree nor disagree*, 5 = *moderately agree*, 6 = *agree*, 7 = *strongly agree*).

### Procedural Justice (Taken From Gobena and van Dijke, 2016, Who Adapted the Scale From Colquitt, 2001)

“The following items refer to the procedures used to arrive at tax-related decisions.

(1) I have been able to express my views and feelings during those procedures.
(2) I have had influence over the (outcomes) arrived at by those procedures.
(3) Those procedures have been applied consistently.
(4) Those procedures have been free of bias.
(5) Those procedures have been based on accurate information.
(6) I have been able to appeal the (outcomes) arrived at by those procedures.
(7) Those procedures have upheld ethical and moral standards.”

### Distributive Justice (Verboon and van Dijke, 2007)

(1) “The use I make of all kinds of social services reflects in a proper way the taxes I pay.
(2) Regarding social services I get little return for my tax money; reverse coded.
(3) Some groups in society benefit more from the tax system than I do; reverse coded.
(4) I think it is not fair that some people pay less tax than me while they benefit equally from all amenities; reverse coded.
(5) I find that I have to pay too much tax; reverse coded.”

### Power of Tax Authority (Kastlunger et al., 2013)

(1) “Tax evasion is detected in a high percentage of the cases.
(2) Tax authorities combat tax crimes in an efficient way.
(3) Tax evasion is likely to be detected.
(4) Tax authorities control frequently and profoundly.
(5) Due to their knowledge and competence, tax authorities are able to detect quite every act of tax evasion.”

### Voluntary Tax Compliance (Kirchler and Wahl, 2010)

“When I pay my taxes as required by the Ethiopian tax laws and regulations, I do so…

(1) …because to me it’s obvious that this is what you do.
(2) … to support the state and other citizens.
(3) … because I like to contribute to everyone’s good.
(4) … because for me it’s the natural thing to do.
(5) … because I regard it as my duty as a citizen.”

### Enforced Tax Compliance (Kirchler and Wahl, 2010)

“When I pay my taxes as required by the Ethiopian tax laws and regulations, I do so….

(1) …because a great many tax audits are carried out.
(2) … because the tax authority often carries out audits.
(3) … because I know that I will be audited.
(4) … because the punishments for tax evasion are very severe.
(5) … because I do not know exactly how to evade taxes without attracting attention.”
