# Supplementary material for: Make Me Want to Pay. A Three-Way Interaction Between Procedural Justice, Distributive Justice, and Power on Voluntary Tax Compliance
Source: Front Psychol. 2019 Jul 12;10:1632. doi: 10.3389/fpsyg.2019.01632 (PMC6639977; doi:10.3389/fpsyg.2019.01632)
Supplement: Supplementary file 1 [file Data_Sheet_1.ZIP › Data statement.docx]

In addition to the scales reported in the paper, in both studies we also measured the following variables, for projects unrelated to the project reported here:

1. Personal and social norms (Wenzel, 2004).
2. Identification with the nation (Tyler & Blader, 2001).
3. Just world beliefs (Lipkus, 1991).
4. Overall justice judgments (Ambrose & Schminke, 2009).
5. Sanction severity (Verboon & van Dijke, 2007).

In addition, in Study 2 we also measured:

1. Coercion (Kastlunger et al., 2013).
2. Trust (Mayer & Gavin, 2005; McAllister, 1995).

In Study 1 we measured voluntary tax compliance with a 10-item scale and enforced tax compliance with an 8-item scale (Kastlunger et al., 2013). Following conventions in the literature (e.g., Kastlunger et al., 2013; Kirchler & Wahl, 2010; Kogler, Batrancea, Nichita, Pantya, Belianin, & Kirchler, 2013) we used only five items of each scale. Results as reported in the main text do not change in any meaningful way when the full scales are used.

None of the data on these additional variables from these two data sets will be used for future publications.

**References not in the paper**

Ambrose, M. L., & Schminke, M. (2009). The role of overall justice judgments in organizational justice research: A test of mediation. *Journal of applied psychology, 94*, 491-500.

Kogler, C., Batrancea, L., Nichita, A., Pantya, J., Belianin, A., & Kirchler, E. (2013). Trust and power as determinants of tax compliance: Testing the assumptions of the slippery slope framework in Austria, Hungary, Romania and Russia. *Journal of Economic Psychology, 34,* 169-180.

Lipkus, I. (1991). The construction and preliminary validation of a global belief in a just world scale and the exploratory analysis of the multidimensional belief in a just world scale. *Personality and Individual Differences, 12*, 1171-1178.

Mayer, R. C., & Gavin, M. B. (2005). Trust in management and performance: who minds the shop while the employees watch the boss?. *Academy of management journal, 48*, 874-888.

McAllister, D. J. (1995). Affect-and cognition-based trust as foundations for interpersonal cooperation in organizations. *Academy of management journal, 38*, 24-59.

Tyler, T. R., & Blader, S. L. (2001). Identity and cooperative behavior in groups. *Group Processes & Intergroup Relations, 4*, 207–226.

Wenzel, M. (2004). The social side of sanctions: Personal and social norms as moderators of deterrence. *Law and human behavior, 28*, 547-567.
